# Single‐Cell Identification of Bacterivorous Mixotrophs in a Freshwater Habitat

**DOI:** 10.1111/1758-2229.70456

**Published:** 2026-11-09

**Authors:** Ying Wang, Fernanda Henderikx‐Freitas, Qian Li

**Affiliations:** ^1^ School of Oceanography, Shanghai Jiao Tong University Shanghai China; ^2^ Department of Oceanography University of Hawaii at Manoa Honolulu Hawaii USA

## Abstract

The nutritional strategy of phago‐mixotrophy among pigmented eukaryotes has been widely recognized and characterized across various taxonomic lineages. While their ecological role as bacterivores in different aquatic environments is acknowledged, establishing a clear connection between phago‐mixotrophic activities and taxonomy within natural communities remains challenging. In this study, we utilized a combination of microscopy and fluorescence‐activated single‐cell sorting techniques to investigate the diversity and temporal variations of bacteria‐feeding mixotrophs in a freshwater habitat. We identified four groups of mixotrophs using both methods: Pedinellales (Dictyochophyceae), *Cryptomonas* (Cryptophyceae), *Dinobryon* (Chrysophyceae) and a single‐chloroplast bearing group within Chlorophyta. Together, these groups accounted for 58%–96% and 54%–95% of total mixotrophs detected by microscopy and cell sorting, respectively. Samples collected in different seasons demonstrated that Pedinellales and Chlorophyta were more abundant in spring and summer/autumn, while *Dinobryon* was only present in winter when temperatures were lowest and light intensity was moderate. Our findings, with 6 classes and 12 genera identified as bacterivorous mixotrophs, highlight the promising potential of single‐cell techniques in aquatic microbiology. This approach reveals the taxonomic identity and feeding activities of phago‐mixotrophs in situ, thereby enhancing our understanding of their functional ecological roles within microbial food webs.

## Graphical abstract

A workflow diagram showing the taxonomic identification of bacteria‐consuming mixotrophs from natural environments, achieved through fluorescence‐activated single‐cell sorting and microscopic observation. The single‐cell approach allows us to establish a clear connection between biodiversity and functional activities in aquatic environments with high resolution.

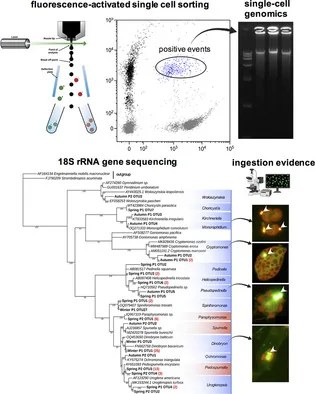

## 1 Introduction

Early studies of pelagic bacterivores, conducted before the 21st century, primarily focused on heterotrophs, including heterotrophic flagellates, ciliates and some amoebas (Sanders et al. 2001; Calbet et al. 2001). As research progressed, it became evident that a broad suite of pigmented eukaryotes, referred to as photo‐phago‐mixotrophs, can also effectively remove bacteria through phagocytosis (Mitra et al. 2016; Stoecker et al. 2017; Li et al. 2022). These predation interactions between bacteria and bacterivorous mixotrophs serve as a vital link between prokaryotes and eukaryotes within microbial food webs. Investigations of the taxonomic composition, abundances and grazing ecophysiology of these mixotrophs will ultimately enhance our understanding of trophic transfer efficiency between bacterial and eukaryotic communities, food web dynamics, as well as carbon and nutrients cycling in various aquatic ecosystems.

The characterization of eukaryotic mixotrophs in aquatic environments is mostly based on laboratory studies that used isolates, demonstrating that marine mixotrophs are commonly associated with various groups from haptophytes (Jones 1994; Legrand 2001; Avrahami and Frada 2020; Koppelle et al. 2022), dinoflagellates (Nygaard and Tobiesen 1993; Jeong et al. 2005), dictyochophytes (Li et al. 2021, 2022), among others. In freshwater systems, mixotrophic bacterivores have primarily been reported among chrysophytes (e.g., *Ochromonas* sp., Sanders et al. 1990; Wilken et al. 2019 and *Dinobryon* sp., Caron et al. 1993; Princiotta et al. 2016) and cryptophytes (e.g., *Cryptomonas* sp., Costa et al. 2022). Chlorophytes have traditionally been considered primarily phototrophic or, under some conditions, capable of osmotrophy, absorbing dissolved organic carbon to enhance growth and biomass productivity (Candido and Lombardi 2020; Proietti Tocca et al. 2024). Recent studies, however, indicate that certain chlorophytes also exhibit phagotrophic behaviour, ingesting small particles or bacteria, indicating mixotrophy may be more widespread among Chlorophyta species than previously recognized (Anderson et al. 2018; Bock et al. 2021; Pang et al. 2022; Charvet et al. 2024).

Identifying bacterivorous mixotrophs directly in natural environments remains challenging for several reasons. Firstly, mixotrophic lineages are highly diverse and phagotrophic capability can vary considerably among closely related species. For instance, a study of *Micromonas pusilla* isolated from the Arctic Ocean suggested mixotrophy (Mckie‐Krisberg and Sanders 2014), while another *Micromonas* species (*Micromonas polaris*) displayed no evidence of feeding during laboratory studies (Jimenez et al. 2021). Similar patterns are observed in *Alexandrium*, where some species are active mixotrophs while others lack this ability (Blossom and Hansen 2021; Lim et al. 2019) and in *Ochromonas*, which spans from obligate to facultative mixotrophy (Lie et al. 2018; Wilken et al. 2019). Secondly, research indicates that the grazing behaviours of mixotrophs are versatile and highly adaptable. These behaviours can change significantly depending on environmental conditions such as nutrient availability, light, temperature and prey abundance (Edwards 2019; Li et al. 2021; Blossom and Hansen 2021; Princiotta et al. 2023). For example, laboratory studies show that light and temperature can shift the balance between phototrophy and phagotrophy in *Takayama helix* and *Picochlorum* sp. favours phagotrophy at elevated temperatures (Pang et al. 2022). In natural systems, even short‐term meteorological fluctuations can induce rapid changes in bacterivory (Anderson et al. 2018). Taken together, these examples highlight that the diversity and the plasticity of mixotrophy, shaped by both genetic differences and environmental dynamics, contribute to the difficulty of directly identifying mixotrophs and their bacterivorous function in the field.

Accurately distinguishing and identifying mixotrophs within natural communities often requires advanced molecular and enumeration techniques (Beisner et al. 2019). Traditional methodology can be labor‐intensive and generally involves microscopic observation and prey labeling techniques that utilize fluorescence (Mcmanus and Fuhrman 1986; Connell et al. 2020). Other approaches include the application of bromodeoxyuridine (Fay et al. 2013; Millette et al. 2021) and the study of rRNA genes through techniques such as catalysed reporter deposition‐fluorescence in situ hybridization (Unrein et al. 2014; Xiao et al. 2025) and RNA stable isotope probing (Frias‐Lopez et al. 2009; Wilken et al. 2023). Recent research has applied fluorescence‐activated flow cytometry enumeration techniques to retrieve mixotrophs on a community level (Li et al. 2023), as well as sorting techniques designed for higher taxonomic level retrieval of mixotrophs in aquatic environments (Florenza et al. 2024).

The investigation of in situ mixotrophs and their activities, despite facing certain challenges, has been highlighted as a key research priority in the field of aquatic mixotrophy ecology (Millette et al. 2023; Wilken and Mcmanus 2024). In this study, we resolve the taxonomic composition of active mixotrophs at the species level within a freshwater habitat over time. We provide results using both a traditional method integrating grazing experiments with fluorescent prey surrogates and microscopic observation, as well as an emerging technique using fluorescence‐activated flow cytometry single‐cell sorting and sequencing. We observe distinct eukaryotic community compositions in both non‐sorted (the whole community) and sorted water samples (active bacterivores), with temporal variations present in both communities, as fluctuations in temperature and light are expected to strongly shape mixotrophic activities. By resolving the taxonomic composition of active bacterivores at the single‐cell level, this study broadens the application of single‐cell approaches in aquatic ecology and provides a direct link between the phylogenetic and functional diversity of mixotrophic eukaryotes in natural environments.

## 2 Materials and Methods

### 2.1 Study Site and Sample Collection

Water samples were collected from a shallow, small mesotrophic lake located at 31.0° N and 121.4° E in eastern China (Figure 1a) during four sampling periods: April 2023, December 2023, July 2024 and October 2024. For simplicity, these sampling periods are hereafter referred to as spring (April 2023), winter (December 2023), summer (July 2024) and autumn (October 2024), according to their sampling dates. Because each season was represented by a single sampling event, seasonal variation was not a focus of this study and differences among samples are interpreted as temporal variations across the four sampling periods rather than established seasonal patterns.

**FIGURE 1:**
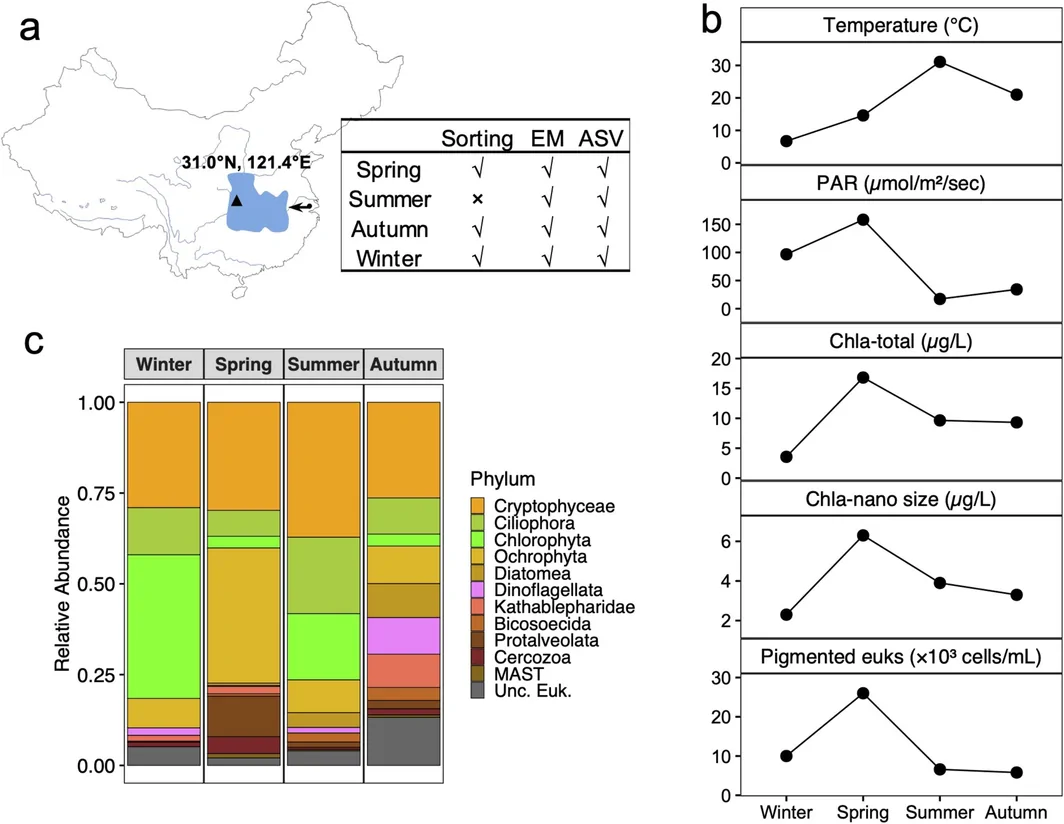
Geographic location of the study site and experimental design for each sampling period (ASV, amplicon sequencing; EM, epifluorescence microscopy; sorting, single‐cell sorting) (a); temperature, photosynthetically available radiation (PAR), chlorophyll a concentration of the total community, chlorophyll a concentration of the nano‐sized eukaryotes and abundance of nano‐sized, pigmented eukaryotes across four seasons/sampling periods (b); phylum‐level community compositions of total eukaryotes (including pigmented and aplastid) revealed by amplicon sequencing (c).

Approximately 2 L water was collected from the surface layer (~1 m), pre‐filtered with a 20 μm nylon sieve, placed into clean transparent bottles and kept in a cooler under dim light to minimize temperature and irradiance fluctuations until arrival at the laboratory (within 1–2 h of collection). The sieve was used to remove microzooplankton grazers and larger plankton that are not expected to graze directly on bacteria. Samples were collected consistently between 10 am and local noon to ensure comparable metabolic activities of eukaryotes.

Environmental parameters that may influence mixotrophic activity were monitored during each sampling. Temperature, salinity, dissolved oxygen (DO) and chlorophyll a concentrations of the total phytoplankton community (including nano‐ and micro‐sizes) were measured at the surface using sensors integrated on a vertical profiler (the YSI Pro DSS, Xylem Inc., USA). Temperature was measured with a platinum resistance thermistor (±0.01°C; YSI Inc 2010), salinity was derived from electrical conductivity using the UNESCO practical salinity scale and DO was determined by an optical luminescence quenching sensor, where oxygen quenches the luminescence of a dye proportionally to DO concentration (detection limit < 0.1 mg L^−1^; YSI Inc 2010). Photosynthetically available radiation (PAR, 400–700 nm) was measured with a QSL‐2100 scalar irradiance sensor (Biospherical Instruments, USA) using a cosine‐corrected silicon photodiode detector. Upon arrival at the lab (within 1 h), approximately 50 mL water from the pre‐filtered bulk sample was aliquoted into smaller bottles to measure dissolved inorganic nitrogen (including nitrate, nitrite and ammonia) and phosphate concentrations using the Auto Discrete Chemical Analyser (AQ 400; Seal Analytical, Germany), following standard colorimetric methods: nitrate was reduced to nitrite on a cadmium–copper column followed by diazotization (absorbance at 540 nm), nitrite directly by diazotization, ammonium by the indophenol blue reaction (absorbance at 630 nm) and phosphate by the molybdenum blue method (absorbance at 880 nm) (Grasshoff et al. 1999). For chlorophyll a analysis of the pico‐ and nano‐sized phytoplankton community, 500 mL of pre‐filtered samples were filtered through a 0.7 μm glass fibre filter (Whatman), pigments were extracted in 90% acetone for 12 h at 4°C in the dark and chlorophyll a was quantified with a Turner Designs fluorometer equipped with an acidification module. This method corrects for phaeopigment interference by comparing fluorescence before and after acidification and concentrations were calculated using the fluorometer calibration protocol and equations from Parsons et al. (1984).

### 2.2 Pico‐ and Nano‐Sized Microbial Abundances In Situ

Following Marie et al. (2014), approximately three 2 mL aliquots were fixed with glutaraldehyde at 0.5% final concentrations (Sigma‐Aldrich), treated with 0.01% final concentration of Pluronic F68 to avoid cell attachments to the plastic tubes, flash frozen in liquid nitrogen and stored with cryovials in −80**°**C. Glutaraldehyde was used because it reliably preserves phytoplankton cell integrity and natural pigment autofluorescence and Pluronic F68 helps reduce cell loss during freezing and storage. These vials were thawed at a later time, vortexed gently and analysed using a CytoFLEX flow cytometer (FCM) (Beckman Coulter, USA) to enumerate pico‐ and nano‐sized pigmented eukaryotes (PE) based on excitation with a 488‐nm blue laser at a medium flow rate (30 μL min^−1^). Heterotrophic flagellates (HF) were enumerated by DAPI staining (Biotium) using an Olympus BX61 epifluorescence microscope as they are difficult to quantify reliably by flow cytometry due to high background fluorescence from bacteria and detritus in our samples (Christaki et al. 2011). PE were quantified using both flow cytometry and microscopy for cross‐validation and counts obtained from each method are reported separately.

### 2.3 Pico‐ and Nano‐Sized Eukaryotic Community Composition

An additional 1 L of pre‐filtered water sample was simultaneously filtered onto a 0.2 μm polycarbonate membrane filter (Whatman) to collect the natural microbiome DNA. Total environmental DNA was extracted using a column‐based, DNeasy PowerSoil Pro Kit (47016) (Qiagen, Germany) and purified using VAHTS DNA Clean Beads (N411‐03, Vazyme, China). Afterwards, the hypervariable V4 region of eukaryotic 18S rRNA genes (~400 bp) was amplified with a universal primer pair of 547F (5′‐CCAGCASCYGCGGTAATTCC‐3′) and V4R (5′‐ACTTTCGTTCTTGATYRA‐3′) (Stoeck et al. 2010). PCR products from each sample were purified and pooled in equal amounts, followed by pair‐end 2 × 250 bp sequencing using the Illumina NovaSeq platform at Shanghai Personalbio Co. Ltd. Quality check and chimeras removal of raw amplicon sequence variants (ASVs) were conducted using DADA2 (Callahan et al. 2016). Filtered ASV sequences were further classified with the SILVA database (Pruesse et al. 2007) using QIIME2 (Bolyen et al. 2019).

ASVs annotated as multi‐cellular organisms (e.g., Vertebrata and Arthropoda) and parasites (e.g., Cryptomycota and Amoeba) that were not the focus of this study were excluded from further analysis. After filtration, a total of 286,840 reads were retrieved from the four seasons, representing eleven classified and one unclassified eukaryotic phyla (Table S1). All partial 18S rRNA gene sequences retrieved from the total community were deposited in GenBank with BioProject number PRJNA1204479.

### 2.4 Grazing Experiments With Single and Multiple‐Prey Types

The grazing experiment was set up immediately after sample collection, referring to our previous study (Li et al. 2023). Briefly, to identify common bacterivores from in situ water samples, approximately 200 mL of pre‐filtered water was amended with 1.0 μm carboxylate‐modified polystyrene yellow‐green fluorescent beads (catalogue #L4655, Sigma Aldrich) at a final concentration of ~2.0 × 10^5^ mL^−1^, accounting for 10%–50% of the in situ bacterial concentrations. Incubation was carried out for 2–24 h under controlled temperature and light conditions simulating the sampling environment across different seasons in a temperature‐ and light‐controlled incubator (GDN‐600, Yanghui Technology, Beijing, China). A minimum incubation time of 2 h was used to ensure sufficient prey ingestion for the recovery of actively feeding cells (Landry and Selph 1995; Li et al. 2021, 2023). Longer incubation periods primarily reflected the availability of the shared flow cytometry facility, where subsequent cell sorting was conducted.

The 1.0 μm beads were chosen as the prey surrogates in our previous study because they retrieved the best fluorescence detection sensitivity on flow cytometry (Li et al. 2023). In the current study, we also conducted multiple‐prey experiments including 1.0 and 0.5 μm polystyrene yellow fluorescent beads (catalogue #L5530, Sigma Aldrich), as well as live *Synechococcus* (strain WH7803) to assess possible feeding preferences or discriminations on the 1.0 μm beads.

Microscopic observations of potential prey discrimination among different bacterivores indicated some variation; however, all tested prey (including 1.0 and 0.5 μm beads and live *Synechococcus*) were able to be effectively ingested by common bacterivores (Figure S1). Consequently, the 1.0 μm beads were chosen as the standardized prey surrogates for all grazing experiments conducted. To minimize cell clumping and prevent surface attachments between cells and beads, Pluronic F‐68 (Sigma Aldrich) was added to the water samples at a final concentration of 0.01% (Marie et al. 2014).

### 2.5 Single Cell Sorting of Active Bacterivores

Unfixed live cells were used for flow cytometry sorting to avoid potential covalent cross linking by glutaraldehyde, which can compromise nucleic acid integrity and reduce amplification efficiency (Douglas and Rogers 1998). Between 2 and 24 h after the grazing experiment incubation, accounting for sample transport, instrument preparation and time for grazing to occur, 1–2 mL unfixed, unstained aliquots were subjected to FCM FACS Aria II (Beckman Dickinson, USA) for single‐cell sorting of actively feeding bacterivores. Bacterivores including mixotrophs and heterotrophs, were gated together based on high green fluorescence excited from the ingested beads (800–50,000 a.u. of FITC channel with ~400 V gain). A threshold between higher red fluorescence (> 500 a.u. of PerCP‐Cy5.5 channel with ~300 voltage gain) and lower red fluorescence (100–500 a.u. of PerCP‐Cy5.5 channel with ~300 voltage gain) was further applied to increase qualitative separation between putative mixotrophs (higher fluorescence) and heterotrophs (lower fluorescence).

Active bacterivores were targeted randomly across the entire gating area and sorted as single‐cells into 96‐well plates prefilled with 1 μL PBS buffer (one cell per well), at a 10 μL/min sample flow rate. Approximately 50 cells per sampling period were targeted as a practical sorting effort, considering the relatively low abundance of some actively feeding populations and the time required for single‐cell sorting and subsequent processing. No cells were sorted during the summer campaign, while a total of 160 single cells were retrieved from the other three sampling periods. Immediately after sorting, the plate was sealed and stored at −80°C until further processing.

### 2.6 Single‐Cell Genomic Amplification

Sorted cells were thawed, following a lysis/denaturation and isothermal genomic amplification reaction using the DLB buffer of REPLI‐g Single Cell Kit (Qiagen, catalogue #150343). The kit uses REPLI‐g sc Polymerase, an optimized formulation of the high‐fidelity enzyme Phi 29 polymerase, to amplify complex genomic DNA using multiple displacement amplification (MDA) technology that provided highly uniform amplification across the entire genome. Though primarily used with mammal cells and bacteria, a few studies have successfully used it in marine protists, including marine taxa (Chen et al. 2021; Wilken, Yung, et al. 2019) and freshwater mixotrophs (Florenza et al. 2024). The isothermal strand‐displacing reaction was performed following the product instruction. Specifically, the lysis and denaturation reaction (with 3 μL pre‐prepared lysis buffer) was incubated at 65°C for 10 min, then inactivated by adding 3 μL Stop Solution. The denaturant DNA material was afterward mixed with 29 L REPLI‐g sc Reaction Buffer (contains primers, dNTP and salt), 9 μL H_2_O and 2 μL REPLI‐g sc Polymerase, incubated for 8 h at 30°C, followed by a stop step by decreasing the temperature to 65°C for 5 min.

### 2.7 18S rRNA Genes Phylogeny

Amplified genomic DNA (gDNA) was diluted at 100× and used as the downstream PCR amplification template. The near‐full length 18S rRNA gene of eukaryotes was amplified by PCR with the TaqMan polymerase PCR System (Mei5bio, MF848) using forward primer Euk63F 5′‐ACGCTTGTCTCAAAGATTA‐3 and Euk1818R 5′‐ACGGAAACCTTGTTACGA‐3′ (Lepere et al. 2011). Amplicons were purified and sequenced (Sanger) using the same forward PCR primer of Euk63F, yielding an overall gene sequence length of 840–900 bp.

For phylogenetic analyses, sequences with ≥ 98% similarity were grouped into the same operational taxonomic units (OTUs). Homologous reference sequences were then retrieved from the NCBI GenBank based on BLAST similarity, with additional sequences from certain neighbour genera. Multiple sequence alignments including 22 OTUs, 18 homologous references and 10 additional taxa were performed with MAFFT v7.450 using the G‐INS‐i algorithm in Geneious R11.1.5. Terminal sites that lacked data for any of the sequences were trimmed and any sites with greater than 0.5% gaps were removed from the alignment, which generated a final sequence length of 837 bases. Phylogenetic trees were constructed using MrBayes v3.2.6 in Geneious R11.1.5 (Huelsenbeck and Ronquist 2001), implementing the GTR substitution model and the CAT approximation of rate heterogeneity, supported by two runs of four chains for 1,000,000 generations which were subsampled every 200 generations. The Bayesian majority consensus tree was further edited within iTOL (Letunic and Bork 2019).

All partial 18S rRNA gene sequences retrieved from cell sorting were deposited in GenBank with accession numbers PV348919–PV348940.

### 2.8 Microscopic Observation

One hour post each of the grazing experiment, 10–20 mL water aliquots were fixed with glutaraldehyde (0.5% final concentration), stained with DAPI (Biotium) and filtered onto a 2.0 μm pore size polycarbonate membrane filter (Whatman). Filters were mounted on glass slides and scanned under epifluorescence microscopy (Olympus 61X) with illumination settings for DAPI (Ex. 405 nm, Em. 400–480 nm), chlorophyll (Ex. 450 nm, Em. 575‐IF), prey (Ex. 545 nm, Em. 605/55 nm) and a longpass filter BA575‐IF for all fluorescence between green‐yellow and red.

Nano‐sized eukaryotic cells that contain one to six inherited, regular‐shaped chloroplasts were categorized as pigmented eukaryotes and those with ingested prey were counted as mixotrophs or mixotrophic bacterivores. Four populations at the genus or order level that harbour different numbers and shapes of chloroplasts were identified based on their unique morphology and characteristics referring to various literature, including *Dinobryon* (Stoecker et al. 2017; Jeong et al. 2023), *Cryptomonas* (Hoef‐Emden and Melkonian 2003), *Pedinellales* (Gerea et al. 2016; Koppelle et al. 2024; Florenza et al. 2024) and Chlorophyta (L. Silva 1999 and book chapters of Kristiansen and Andersen 1986; Wehr et al. 2015).

These identified genera include *Dinobryon* belonging to Chrysophyceae (protoplasts housed in a cylindrical to funnel‐shaped lorica with a stigma), Pedinellales belonging to Dictyochophyceae (three to six chloroplasts located at the periphery of cells), *Cryptomonas* belonging to Cryptophyceae (hexagonal periplast plates and phycobilin‐emitted orange fluorescence) and parietal single‐chloroplast‐bearing Chlorophyta with common chloroplast shapes resembling the moon, horseshoes, cups, etc. The remaining mixotrophic bacterivores were classified as ‘Other mixotrophs’, likely including mixotrophs from other species of Ochrophyta, Dinoflagellata, Chlorophyta and Cryptophyceae. Inherited‐chloroplast‐absent nano‐sized flagellates were all classified as heterotrophs as species identification based on morphology is complex and those with ingested prey were counted as heterotrophic bacterivores.

## 3 Results

### 3.1 Environmental and Biological Characteristics of the Study Site

Environmental conditions varied among sampling periods (Table 1; Figure 1b). Water temperature ranged from 6.7°C to 31.1°C, while underwater light intensity varied between 17.3 and 177.3 μmol m^−2^ s^−1^ PAR, with lower light availability associated with shading by aquatic macrophytes. Nitrate and nitrite concentrations ranged from 2.0 to 39.5 μmol L^−1^, whereas ammonia remained low (< 0.1 μmol L^−1^) except during one sampling period (13.3 μmol L^−1^).

**TABLE 1:** Physical and biochemical characteristics of the study site.

Season	PAR (μmol m^−2^ s^−1^)	Temp (°C)	NO_3_ + NO_2_ (μmol L^−1^)	NH_4_ (μmol L^−1^)	Chl a‐Total (μg L^−1^)	Chl a‐Pico and Nano (μg L^−1^)	PE^a^ (cells mL^−1^)	PE^b^ (cells mL^−1^)	HF^b^ (cells mL^−1^)
Winter	96.7	6.7	19.6	13.3	3.6	2.3	8.78E+03	1.0E+04	6.2E+03
Spring	158.2	14.6	NA	NA	16.8	6.3	2.6E+04	1.50E+04	4.6E+03
Summer	17.3	31.1	39.5	0.1	9.7	3.9	6.9E+03	5.2E+03	6.8E+03
Autumn	34.3	21.0	2.5	0.07	9.3	3.3	5.24E+03	4.9E+03	6.1E+03

Abbreviations: Chl a‐Pico and Nano, Chl a of Pico‐ and Nano‐sized eukaryote; Chl a‐Total, Chl a of total community; HF, heterotrophic flagellates; PAR, photosynthetically available radiation; PE, pigmented eukaryotes; Temp, temperature.

^a^ Results were derived from flow cytometry analysis.

^b^ Results were derived from microscopic observations.

Chlorophyll a concentrations and the abundance of pico‐ and nano‐sized pigmented eukaryotes (PE) varied among sampling periods (Table 1; Figure 1b). PE abundance ranged from 0.7 × 10^4^ to 2.6 × 10^4^ cells mL^−1^ and generally followed changes in the pico‐ and nano‐sized chlorophyll a fraction, although periods with relatively low chlorophyll a (winter 2023) still supported substantial PE abundances, suggesting shifts in community composition (Table S1). Heterotrophic flagellates (HF) were comparatively stable, ranging from 4.6 × 10^3^ to 6.8 × 10^3^ cells mL^−1^ (Table 1).

Amplicon sequencing showed that the pigmented eukaryotic community was primarily composed of Cryptophyceae, Chlorophyta, Ochrophyta, Bacillariophyta and Dinoflagellata, whereas heterotrophic taxa were dominated by Ciliophora, Kathablepharidae, Bicosoecida, Cercozoa, MAST and Protalveolata (Figure 1c). Based on 18S rRNA gene abundance, Cryptophyceae, consistently dominated by *Cryptomonas*, represented the most abundant pigmented lineage throughout the study. Chlorophyta was the second most abundant group and was represented primarily by *Picochlorum* and unclassified Chlorophyceae (Table S1).

### 3.2 Single‐Cell Sorting Efficiency

The active bacterivore populations with high green fluorescence excited from ingested prey on FCM showed different shapes among different seasons. The most distinguishable and intact cloud of feeding bacterivores was observed from the winter sample (Figure 2a). The spring and autumn bacterivore populations presented similar distribution shapes (Figure 2b,c). With the BD FACS Aria II sorter, we sorted a total of 160 single cells from three different seasons (winter, spring and autumn) (Figure 2b). Across all sorted samples, 140 cells were successfully amplified, yielding genomic DNA fragments exceeding 15 kb. Among these, 112 cells yielded positive 18S rRNA gene amplification (Figure 2d).

**FIGURE 2:**
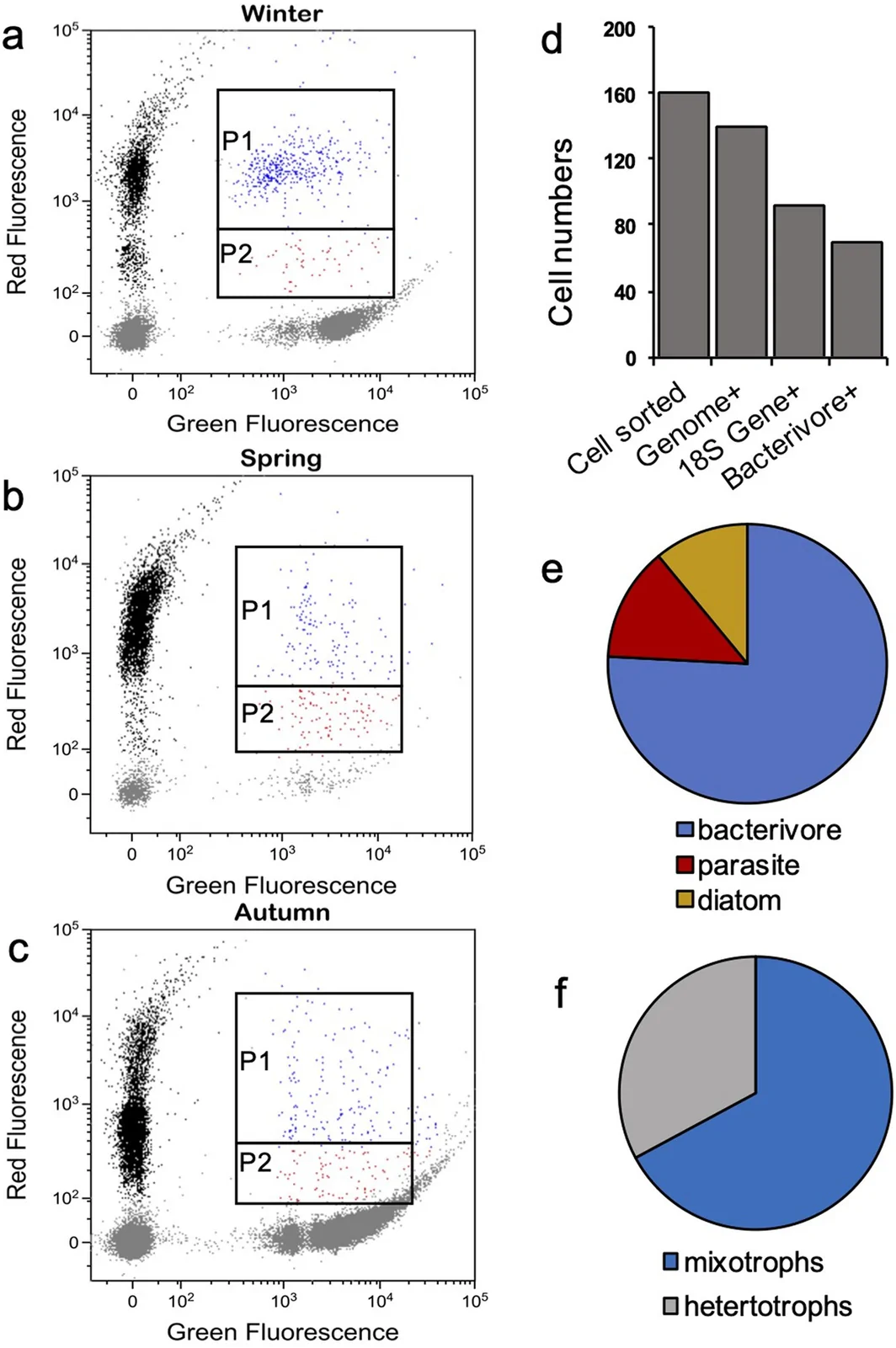
Flow cytometry‐based bacterivore populations gated for sorting putative mixotrophs (P1, events shown in blue) and putative heterotrophs (P2, events shown in red) with samples from winter (a), spring (b) and autumn (c). Panel d demonstrates total events numbers from the initial sorting, samples recovered positive genomic amplifications, positive 18S rRNA genes and annotated to be putative bacterivores. Panels e and f present community compositions in samples recovered with positive 18S rRNA genes and annotated to be bacterivores, respectively.

Of the 112 eukaryotic cells, 70 were identified as actively feeding bacterivores commonly found in aquatic environments, including 47 mixotrophs and 23 heterotrophs. The rest of the amplified 18S rRNA genes were affiliated with parasitic dinoflagellates and diatoms, which were not considered bacterivores and excluded from further analysis (Table S2; Figure 2e,f). Consequently, our single‐cell sorting retrieved a mean final success rate (number of identified active bacterivores: total number of sorted single events) of 44% across all samples. Sorting efficiency varied across seasons, possibly associated with community composition and bacterivorous activities. Specifically, spring presented the highest final success rate of 73%, followed by winter at 52% (19% false positive results from parasites) and autumn at only 24% (26% false positive results from diatoms).

Approximately 94% of all sorted mixotrophs were retrieved from the higher‐chlorophyll P1 population on FCM and the other 6% were retrieved from the lower‐chlorophyll P2 population on FCM (Figures 2, 3; Table S2). In comparison, 61% of sorted heterotrophs were from the P2 population and the remaining 39% were from the P1 population. Overall, the higher‐chlorophyll P1 population revealed 83% mixotrophs and the P2 population retrieved > 90% heterotrophic bacterivores.

**FIGURE 3:**
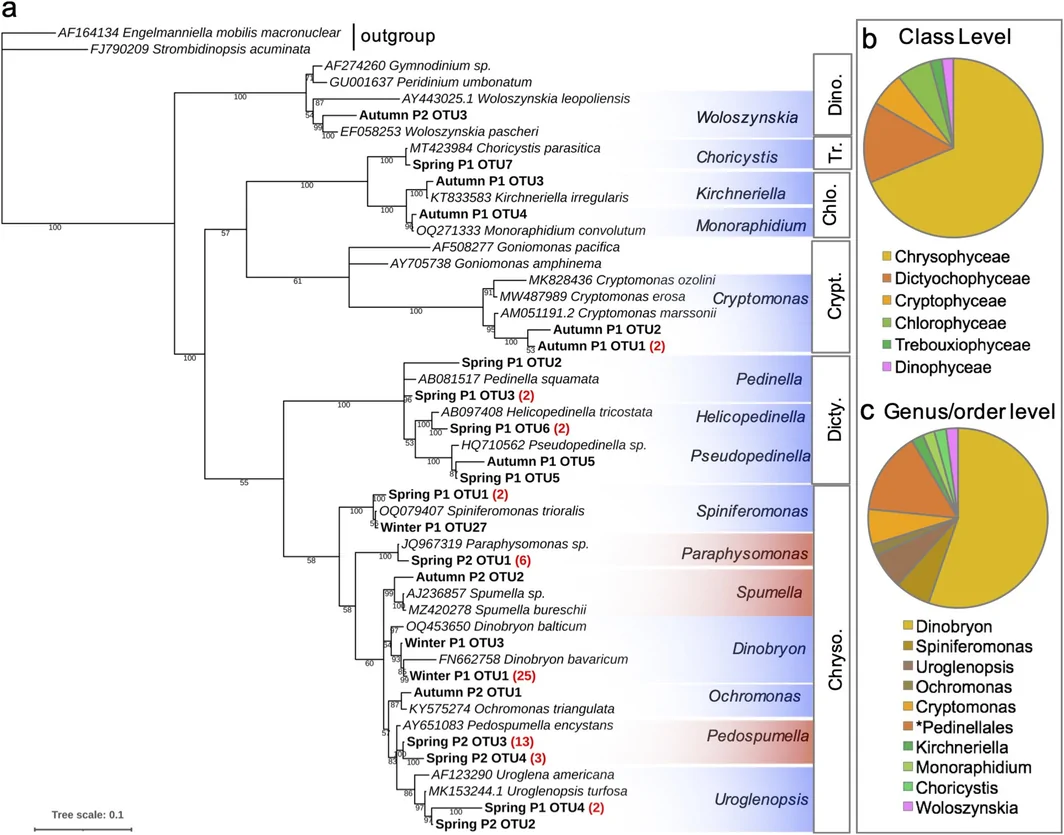
Neighbour‐joining phylogenetic tree of sorted bacterivores constructed using partial 18S rRNA genes (a) and their taxonomic compositions on class level (b) and genus or order level (c) presented in pie charts. OTUs of bacterivores belonging to each genus in panel a were noted as Season_population gate on FCM_OTU#, followed by the total number of each OTU retrieved in a bracket if it was more than one. Chlo., (Chlorophyceae); Chryso., Chrysophyceae; Crypt., (Cryptophyceae); Dicty., Dictyochophyceae; Dino., Dinophyceae; Tr. (Trebouxiophyceae). *Pedinellales was the only order‐level group that included three genera of *Pedinella*, *Pseudopedinella* and *Helicopedinella*. All OTU sequences are listed in Table S2.

### 3.3 Taxonomic Composition of Bacterivores From Sorted Cells

The 70 successfully sorted and annotated bacterivores revealed 22 OTUs, belonging to 12 genera of mixotrophs and 3 genera of heterotrophs that were associated with six classes (Figure 3). All 23 heterotrophic sequences (4 OTUs) were affiliated with Chrysophyceae, composed of *Pedospumella*, *Spumella* and *Paraphysomonas*. Focusing on only mixotrophs, Chrysophyceae accounted for 69% of total sorted bacterivores on the class level (Figure 3b). The second dominant class is Dictyochophyceae, contributing 15% of total sorted mixotrophic bacterivores. The remaining four classes, Cryptophyceae, Chlorophyceae, Trebouxiophyceae and Dinophyceae, contributed 2%–6% of total sorted bacterivores.

On genus or order level, the top two groups were *Dinobryon* of Chrysophyceae and Pedinellales of Dictyochophyceae, accounting for 55% and 15% of total bacterivores, respectively (Figure 3c). The remaining genera, including *Spiniferomonas, Uroglenopsis* and *Ochromonas* of Chrysophyceae, *Pedinella*, *Pseudopedinella* and *Helicopedinella* of Dictyochophyceae, *Monoraphidium* and *Kirchneriella* of Chlorophyceae, *Choricystis* of Trebouxiophyceae, *Cryptomonas* of Cryptophyceae and *Woloszynskia* of Dinophyceae, contributed only between 2% and 6% of the total community. Given the relatively small number of successfully recovered bacterivores (*n* = 70), the sorted populations likely represent only a subset of the actively feeding bacterivore community. Therefore, the absence of a particular taxon from a sampling period should be interpreted as its absence from the recovered sorted population rather than its ecological absence from the community.

### 3.4 Taxonomic Composition of Bacterivores Observed by Microscopy

Mixotrophic bacterivores were identified under epi‐fluorescent microscopy from grazing experiments using single‐ and multiple‐prey types, distinguished by their characteristic morphology and chloroplasts (see details in Section 2.8) (Figure 4a–g; Figure S1). These identified actively feeding mixotrophs included *Dinobryon*‐like (Chrysophyceae), Pedinellales‐like (Dictyochophyceae), *Cryptomonas*‐like (Cryptophyceae) and the single chloroplast‐bearing group of Chlorophyta (Figure 4h). Referring to the amplicon sequence and sorted bacterivore results, microscopy‐identified single‐chloroplast‐bearing Chlorophyta mixotrophs were possibly associated with Chlorophyceae (such as *Kirchneriella* and *Monoraphidium*) and Trebouxiophyceae (such as *Choricystis*). The remaining mixotrophic bacterivores were classified as ‘Other mixotrophs’, possibly including mixotrophs from other populations of Ochrophyta (Chrysophyceae and Dictyochophyceae), Chlorophyta (Chlorophyceae, Trebouxiophyceae and Mamiellophyceae), Dinophyceae and Cryptophyceae (Figure 4h).

**FIGURE 4:**
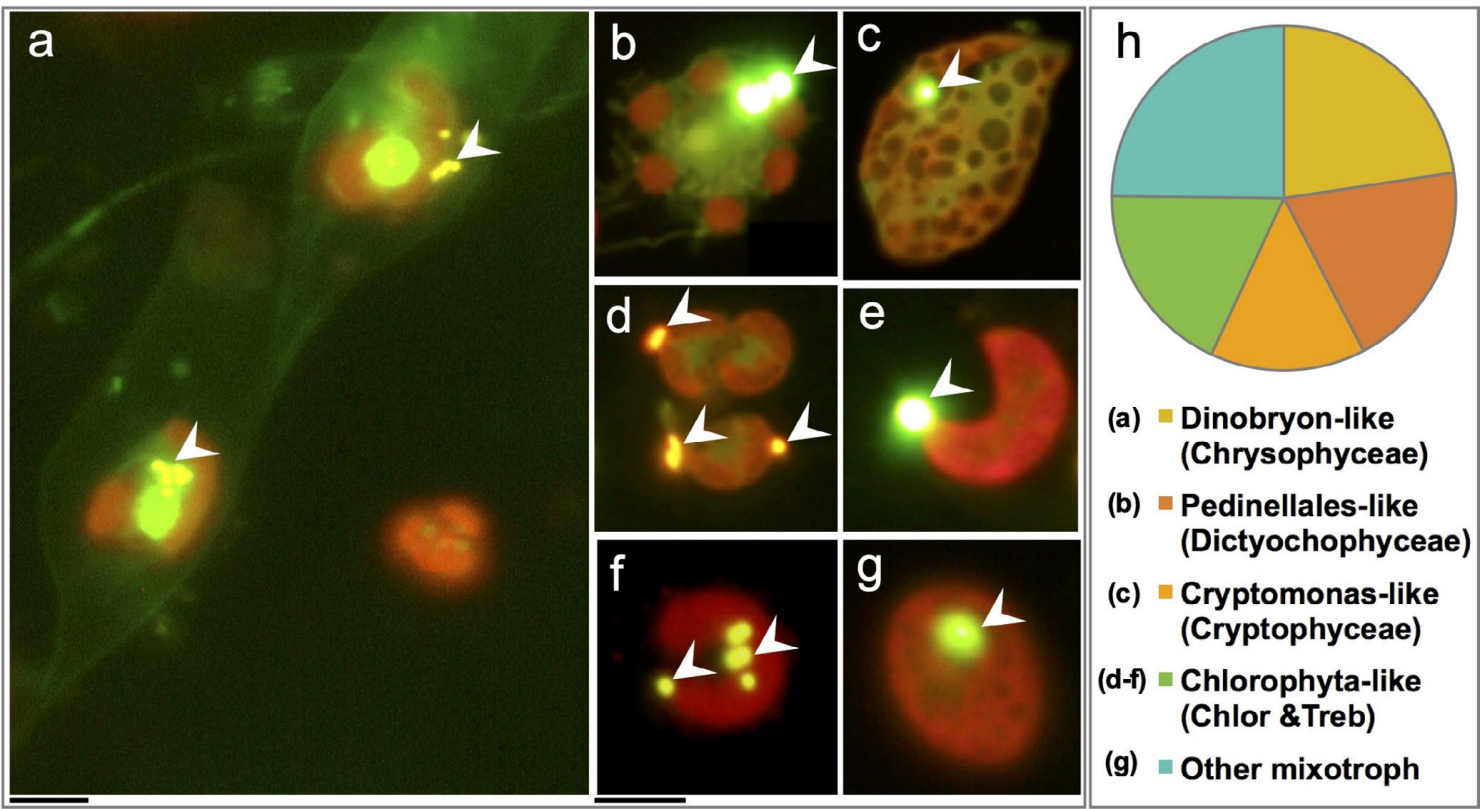
The five microscopy‐identified mixotroph groups based on their distinguishable chloroplasts and morphology characters, including *Dinobryon*‐like (a), Pedinellales‐like (b), *Cryptomonas*‐like (c), (single‐parietal chloroplast‐bearing) Chlorophyta‐like (d–f) and ‘Other mixotrophs’ (g). Arrows denote ingestion evidence of various prey types (0.5 μm beads in a and f, *Synechococcus* in d and all the rest were 1.0 μm beads). Proportions of each group across four sampling periods were shown in panel h. All scale bars are 5 μm in length; images b–g share the same scale bar beneath image f and image a has its scale bar beneath image a. Legend of taxonomy in panel h was organized as genera/order (class); Chlor and Treb refer to Chlorophyceae and Trebouxiophyceae, respectively.

Among the five mixotrophic groups across different seasons, *Dinobryon*, ‘Other mixotrophs’ and Chlorophyta contributed 25%, 23% and 18% of total bacterivores, which were moderately higher than the other three groups. Among Pedinellales and *Cryptomonas*, each group accounted for 20% and 15% of all observed bacterivores. Given that ‘Other mixotrophs’ could encompass a much higher species diversity, those identified four mixotrophic groups were likely abundant or common bacterivores across four seasons. All heterotrophic bacterivores were categorized as one single group of heterotrophs and they contributed a similar proportion to total bacterivores across all seasons to mixotrophs (data not shown).

### 3.5 Temporal Composition of Bacterivorous Mixotrophs Derived From Two Methods

All four identified actively feeding bacterivore groups, observed microscopically, were also present in the sorted bacterivore communities, enabling a comparative analysis between the two approaches (Figure 5a,b). Although the diversity of bacterivores retrieved through cell sorting was lower, partly due to the relatively small sample size of sorted cells (e.g., 160), the majority of common bacterivores were identified by both methods. This was particularly evident in the autumn sample, where both methodologies identified the same four mixotrophic populations of single‐chloroplast bearing Chlorophyta‐like, *Cryptomonas*‐like, Pedinellales‐like and ‘Other mixotrophs’ (including other taxa from Chrysophyceae, Dinophyceae, Chlorophyceae, Trebouxiophyceae, Mamiellophyceae, Cryptophyceae and Dictyochophyceae). In contrast, while Chlorophyta‐like and *Cryptomonas*‐like bacterivores were present in the microscopic samples collected during winter and spring, they were absent in the sorted community.

**FIGURE 5:**
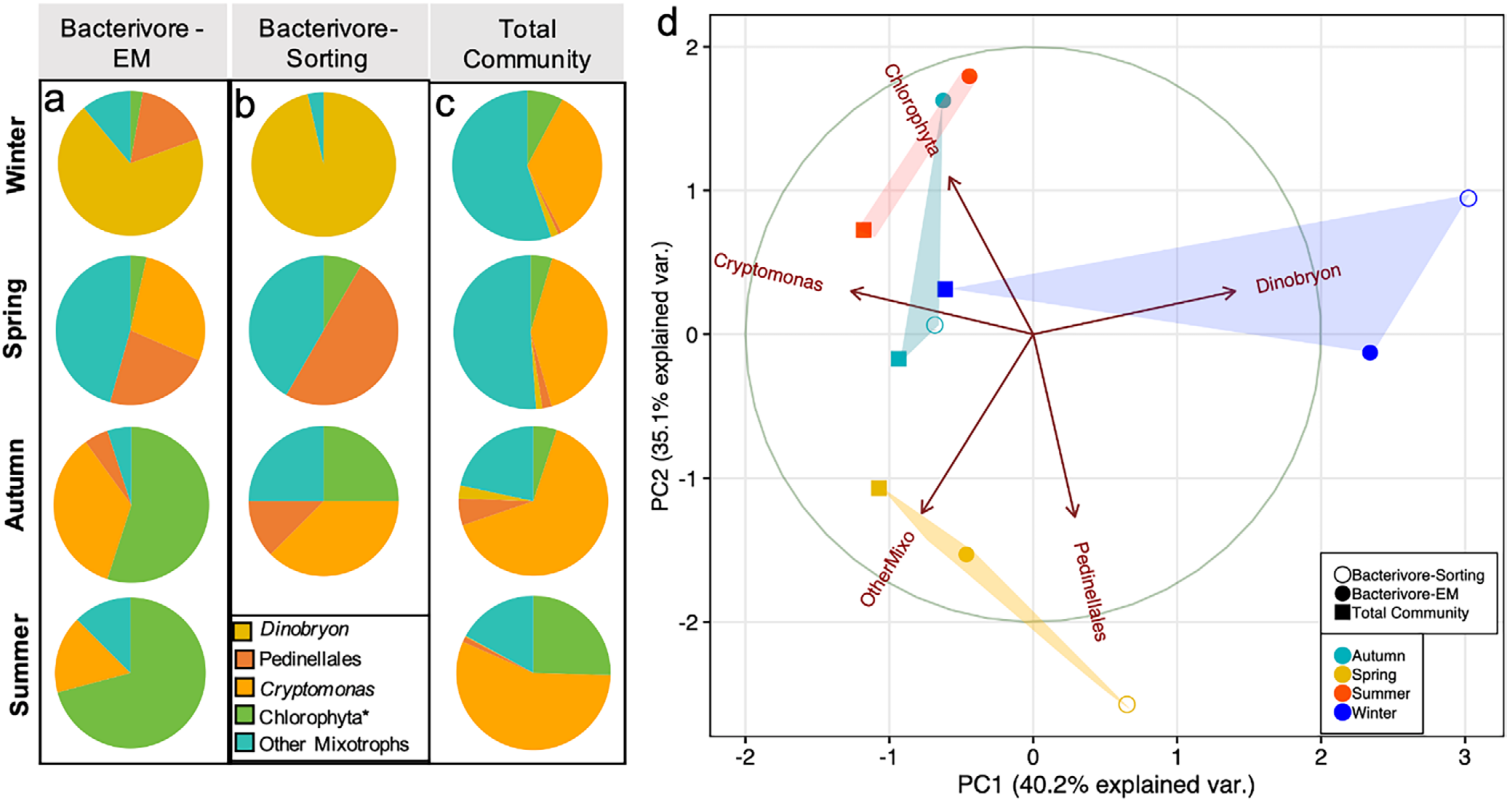
Taxonomic composition of five mixotrophic bacterivore groups obtained from epifluorescence microscopy (EM) (a) and sorting approach (b) of each sampling season; (c) the relative abundances of the five groups in panels a and b in the total pigmented eukaryotic community (gene abundances of amplicon sequencing). (d) principal component analysis (Euclidean distances) among all 11 samples in panels a–c. The circle in panel d denotes the PCA confidence ellipses and samples from the same season were colour‐coded with shades. *A small portion of Chlorophyta taxa from the ASVs that likely contain more than one chloroplast were excluded from the analysis.

The bacterivore communities obtained through both methods demonstrated temporal variations in composition, alongside differences when comparing to their relative abundances among the total pigmented‐eukaryotic community (Figure 5a–c). In the winter sample, *Dinobryon* emerged as the predominant bacterivore, detectable under microscopy and representing the sole group identified through flow cytometry. The spring sample featured notable presences of Pedinellales and ‘Other mixotrophs’ in both sorted and microscopic analyses. Within the autumn samples, both Chlorophyta and *Cryptomonas* were abundant across two methodologies. The summer composition exhibited similarities to that of autumn, with Chlorophyta identified as the most abundant actively feeding bacterivores according to microscopic observations.

Principal component analysis (PCA) revealed similar temporal patterns of bacterivore community composition, with summer and autumn communities showing similar composition associated with Chlorophyta, while winter and spring communities exhibited distinct composition dominated by *Dinobryon*, *Pedinellales* and ‘Other mixotrophs’, respectively (Figure 5d). Specifically, the bacterivore community identified by microscopy in summer and autumn was positioned close to one another, exhibiting a strong correlation with Chlorophyta. Conversely, the winter and spring bacterivore communities were positioned further away, aligning more closely with *Dinobryon* and Pedinellales and ‘Other mixotrophs’, respectively. Sorted bacterivores in autumn and the total communities in summer, autumn and winter displayed overlapping patterns with *Cryptomonas*.

When heterotrophs were incorporated into the bacterivore community analysis, temporal differences among the four sampling periods were observed, with the winter bacterivores showing the greatest separation from the other sampling periods (Figure S2). Microscopy‐identified ingestion observations further showed that bacterivorous mixotrophs represented 22%, 24% and 34% of the < 20 μm pigmented eukaryotes community in winter, spring and summer, respectively, whereas bacterivorous heterotrophic flagellates represented 66%–78% of the corresponding heterotrophic flagellate community. These results indicate a higher prevalence of bacterivory among heterotrophic eukaryotes, together with a non‐negligible and temporally variable contribution from pigmented phago‐mixotrophs.

Comparisons with the total eukaryotic community showed that differences between the identified bacterivore communities and the overall community were generally greater than those between bacterivore communities identified by microscopy and cell sorting (Figure 5a–d). This pattern was also reflected in the PCA ordination, supporting broad agreement between the two approaches while revealing temporal differences in bacterivore community composition (Figure 5d).

## 4 Discussion

### 4.1 Common Bacterivores in the Investigated Freshwater Habitat

Identified actively feeding mixotrophic bacterivores in the investigated freshwater habitat included members from the dictyochophytes, cryptophytes, chrysophytes and chlorophytes. Among the three genera or orders identified through both cell sorting and microscopy, Pedinellales (dictyochophytes), *Cryptomonas* (cryptophytes) and *Dinobryon* (chrysophytes) have all been confirmed to possess phago‐mixotrophic capabilities in various studies (Caron et al. 1993; Urabe et al. 2000; Gerea et al. 2016).

Koppelle et al. (2024) found that Pseudopedinella‐like dictyochophytes and *Cryptomonas*‐like cryptophytes were common bacterivores in a subalpine mountain lake. However, laboratory experiments have reported low ingestion rates in two species of *Cryptomonas* (Tranvik et al. 1989). Additionally, Gerea et al. (2016) demonstrated that Pseudopedinella were widespread across various lakes in higher latitudes of the Southern Hemisphere, contributing significantly to in situ bacterivory rates. In another temperate oligotrophic lake, Pedinellales also appeared to be a dominant mixotrophic bacterivore group (Florenza et al. 2024).

For the single parietal plastid‐bearing chlorophyta (*Kirchneriella* and *Monoraphidium*), the sample numbers in the sorted bacterivore community were low, with only one OTU identified for each genus. Therefore, any interpretations should be made with caution. Nevertheless, these Chlorophyta‐like cells with similar morphological characteristics were commonly observed among bacterivores by microscopy. Based on cell morphology, size and plastid structure (Figure 4d–f), some of the microscopically observed cells may be affiliated with *Kirchneriella* and *Monoraphidium*, consistent with the taxa identified in the sorted populations. Neither population was annotated in the overall community dataset, but it is possible that some of the unclassified Chlorophyceae, which were dominant ASVs during summer and autumn, may share morphological characteristics with these genera. Together, these observations suggest potential bacterivory among these chlorophytes, although further experimental confirmation is required to establish their taxonomic identity and mixotrophic capability.

Previous studies have demonstrated that various marine and freshwater species of chlorophytes possess mixotrophic capability, including members from Chloropicophyceae (Bock et al. 2021), Nephroselmidophyceae (Bock et al. 2021; Bell and Laybourn‐Parry 2003) and Pyramimonadophyceae (Mckie‐Krisberg and Sanders 2014; Anderson et al. 2018), among others. However, none of the three genera associated with putative mixotrophs in this study, *Choricystis*, *Monoraphidium* and *Kirchneriella*, have been experimentally confirmed to exhibit phago‐mixotrophy in laboratory cultures. Our observations therefore suggest that bacterivorous mixotrophy may be more widespread across Chlorophyta than currently recognized, although experimental confirmation of these taxa is needed.

### 4.2 Temporal Variations of Bacterivorous Mixotrophs Were Observed Across Sampling Periods

Differences among the four seasonal sampling periods were observed for the different bacterivore populations identified via both cell sorting and microscopic approaches. For example, Pedinellales, *Cryptomonas* and Chlorophyta were more commonly found in the identified bacterivore communities throughout the year than *Dinobryon*, although their relative contributions varied among sampling periods. These differences coincided with changes in environmental conditions such as light, temperature and nutrients. However, because each season was represented by a single sampling event and multiple environmental variables covaried, the effects of individual environmental factors cannot be resolved from our field observations. Higher‐resolution temporal sampling, together with controlled experiments, will be necessary to distinguish recurring seasonal patterns from shorter‐term variability and to determine the environmental factors regulating bacterivorous mixotrophy in the taxa identified here.

For instance, the relative abundance of *Dinobryon* out of total eukaryotes (based on 18S rRNA gene copy numbers) was relatively consistent and low among sampling periods, ranging from 0.1% in summer to 2% in winter and 3% in autumn (Figure 5c). However, they were only identified as the dominant active bacterivores in the winter samples, accounting for 69% of bacterivores observed by microscopy and 100% of sorted bacterivores. The winter sampling period was characterized by the lowest temperature (~7°C), relatively high irradiance (~100 μmol m^−2^ s^−1^) and differences in several other environmental variables compared with the other sampling periods (Table 1). Using a culture of *Dinobryon sociale*, experimental studies have reported higher ingestion rates at relatively low temperatures (8°C–16°C versus 20°C) and relatively high irradiance (130 versus 66 μmol m^−2^ s^−1^) (Princiotta et al. 2016). Our field observations are therefore consistent with previous experimental studies suggesting that bacterivory may provide an important supplementary nutritional strategy for *Dinobryon* under certain environmental conditions (Kamjunke et al. 2006; Heinze et al. 2013).

Despite being generally ubiquitous bacterivores, the abundances of Pedinellales and *Cryptomonas* differed among sampling periods. Pedinellales were abundant or dominant in both microscopy‐ and sorting‐derived communities during spring, whereas *Cryptomonas* dominated in autumn. These observations coincided with differences in several environmental variables, including temperature, irradiance, chlorophyll a and nutrient conditions, making it difficult to attribute the observed community differences to any single environmental factor. Nevertheless, the prominence of Pedinellales during the spring sampling is consistent with previous findings linking Pedinellales' prevalence to high total chlorophyll levels (Koppelle et al. 2024).

Previous studies also indicate that the trophic strategy of *Cryptomonas* can vary with environmental conditions. For instance, *Cryptomonas* has been reported to function primarily as an autotroph in humic mesotrophic lakes (Tranvik et al. 1989), but can also rely on phagotrophy to supplement its nutrition and energy under low‐light conditions, such as beneath ice in Antarctic lakes during winter (Marshall and Laybourn‐Parry 2002). Given its primarily phototrophic nature, bacterivory may serve as a supplementary nutritional strategy whose activity varies with environmental conditions. Consistent with this trophic flexibility, *Cryptomonas* was abundant in the overall community across sampling periods in our study, as indicated by the amplicon sequencing data, whereas its contribution to the actively feeding bacterivore community varied among sampling periods. The greater bacterivorous activity *Cryptomonas* observed in the autumn/summer samples, which were characterized by relatively low underwater irradiance, is broadly consistent with this physiological flexibility, although our field observations do not establish irradiance as the direct driver (Table 1).

Differences among sampling periods were also observed for the putative mixotrophic Chlorophyta with a single parietal plastid, which represented a greater proportion of the bacterivore community during the summer and autumn sampling periods. Both sampling periods were characterized by relatively low underwater irradiance. Interestingly, physiological experiments under changing light conditions performed with chlorophyte species such as *Picochlorum* (Pang et al. 2022) and *Micromonas* (Mckie‐Krisberg and Sanders 2014) have suggested that bacterivory was enhanced under higher light availability. The apparent difference between these experimental observations and our field pattern may reflect taxon‐specific grazing physiology or the combined influence of multiple environmental variables in natural communities. Further experimental studies are needed to confirm these observations and determine how environmental conditions may influence bacterivory in these chlorophytes.

### 4.3 Strengths and Limitations of the Single‐Cell Sorting Methodology

The average success rate of retrieved bacterivores, defined as the proportion of total sorted events, was approximately 45% across seasons. Of these, ~60% were identified as mixotrophs and ~40% as heterotrophs. Using a similar fluorescence‐activated cell sorting approach, Florenza et al. (2024) reported a higher number and diversity of mixotrophic bacterivores (943 bacterivores out of 1837 sorted events, representing 34 OTUs across six classes) in a temperate oligotrophic lake. Notably, over 80% of their sorted bacterivores belonged to Pedinellales, consistent with our findings and their overall success rate (51%) was comparable to that observed here. Collectively, these results suggest that fluorescence‐activated single‐cell sorting is an effective approach for capturing dominant bacterivores, while also highlighting inherent limitations in resolving the full diversity of mixotrophic taxa in natural aquatic systems.

During the MDA step, approximately 13% of reactions were negative, likely reflecting the absence of a cell in the sorted droplet, unsuccessful deposition, incomplete cell lysis or contamination during single‐cell genomic workflows. Subsequent 18S rRNA amplification failed in an additional ~20% of reactions, likely due to low template representation in the MDA product or primer–template mismatches; systematic PCR failure is unlikely, as other cells from the same batch were successfully amplified. Among the 18S‐positive reactions, 38% did not yield target bacterivores, instead returning sequences from fungi (18%), diatoms (9%) or parasitic dinoflagellates (11%), which were taxa unlikely to be phagotrophic.

These results indicate that the observed failure rates primarily arise from inherent limitations of single‐cell sorting, lysis efficiency and stochastic PCR processes, rather than experimental artefacts. In addition, unintended interactions or attachment with beads other than phagocytosis due to cell surface stickiness (Seymour et al. 2017; Wilken, Choi, et al. 2019) may explain for the sorted diatom and dinoflagellate parasitoid of *Pararosarium* (Table S2), neither of which were seemingly bacterivores. Minimizing cell attachment could potentially be achieved by using non‐ionic surfactants (Marie et al. 2014), reducing incubation time and incorporating microscopy checks (Wilken, Yung, et al. 2019). It is also speculated that the model of the flow cytometry sorter might influence sorting efficiency and enhancements in cell lysis techniques could boost genomic amplification rates, particularly for groups such as chlorophytes with cellulose cell walls and dinoflagellates with thecas.

Our findings indicate that heterotrophs that may also exhibit omnivorous behaviour could be detected with positive chlorophyll fluorescence, such as *Spumella*‐like flagellates (P. C. Silva 1960). When focused on mixotrophs, specificity might be improved by adjusting and refining the gating area for red fluorescence values. Our results further suggested that sorted diatoms are associated with high chlorophyll fluorescence, while aplastid bacterivores often display lower chlorophyll fluorescence.

Beyond taxonomic identification, the single‐cell sorting framework used here also provides opportunities for integration with transcriptomics, proteomics, multiple molecular probes and fluorescent prey tracers, as well as quantitative grazing measurements. Such integration could further link the taxonomic identity of individual cells with their feeding activity, physiological characteristics and molecular responses, providing new opportunities to investigate trophic plasticity under changing environmental conditions.

Collectively, this study applied fluorescence‐activated sorting, combined with single‐cell genomic amplification and microscopic observations, to investigate aquatic mixotrophy, showcasing the potential for uncovering the diversity of actively feeding phago‐mixotrophs in natural water samples at the single‐cell level. Beyond identifying which taxa are capable of bacterivory, our observations of temporal variation in bacterivorous activity among taxa that remained present across sampling periods highlight the potential flexibility of trophic strategies within natural mixotrophic populations. Our findings shed light on the potentially widespread occurrence and functional plasticity of phago‐mixotrophic activity among pigmented eukaryotes in freshwater habitats, providing insights into their roles in mediating elemental cycling between prokaryotes and eukaryotes within aquatic food webs.

## Author Contributions

**Ying Wang:** data curation, investigation, formal analysis, methodology, writing – original draft. **Fernanda Henderikx‐Freitas:** funding acquisition, writing – review and editing. **Qian Li:** conceptualization, methodology, data curation, investigation, formal analysis, supervision, funding acquisition, writing – original draft, writing – review and editing.

## Funding

This work was supported by National Natural Science Foundation of China (42476120, 42106097) and the Ministry of Science and Technology (MOST) ONCE project.

## Conflicts of Interest

The authors declare no conflicts of interest.

## Supporting information


**Figure S1:** Comparison of ingestions by different mixotrophic groups among three prey types, including live *Synechococcus*, 1.0 μm latex beads and 0.5 μm latex beads (a). Results were retrieved and averaged from multi‐prey experiments conducted in different seasons. Observed prey numbers ingested by each mixotroph group were normalized against numbers of ingested 1.0 μm beads. Mixotrophic groups were consistent with those shown in Figure 4. The ‘*x*’ symbol notes the absence of ingestion of *Synechococcus* by *Dinobryon* from observed samples. Panel b shows ingestion evidence from representative cells from each mixotrophic group on different prey types.
**Figure S2:** Pie charts demonstrating the taxonomic composition of all bacterivore groups identified from epifluorescence‐microscopy (EM) (a) and cell‐sorting (b) of each season, including five mixotrophic groups and one heterotrophic group. Panel c presents the relative abundances of those six groups in panels a and b in the total eukaryotic community (gene abundances of amplicon sequencing). Panel d was the principal component analysis plot showing Euclidean distances among all 11 samples in panels a–c. The circle in panel d denotes the PCA confidence ellipses and samples from the same season were colour‐coded with shades. *A small portion of Chlorophyta taxa from the ASVs that likely contain more than one chloroplast were excluded from the analysis.

**Table S1:** Amplicon sequence variants (ASVs) of partial 18S rRNA genes from total eukaryotic communities across four seasons. Datasheets were labelled as ‘season_original data’ and ‘season_filtered data’ which refer to sequences before and after removing non targeting taxa such as parasites and metazoan.

**Table S2:** Taxonomic composition of all sorted events at the OTU level, including targeted bacterivores and non‐bacterivores (text in blue colour). A representative sequence with NCBI Genbank ID from the same OTU was shown in the table and sequence identifiers in the first column were noted as season_population gate_OTU#.

## Data Availability

All genetic sequence data obtained in this study were deposited in the NCBI GenBank (BioProject No. PRJNA1204479 and Gene Accession No. PV348919‐PV348940) (https://www.ncbi.nlm.nih.gov/) and can be obtained from the Supporting Information S1. Environmental variables and experimental results can be obtained from the main text or upon request made to the authors.
